# Study for lightweight finger vein recognition based on a small sample

**DOI:** 10.1038/s41598-024-63002-1

**Published:** 2024-05-25

**Authors:** Yi Ding, Kai Wang, Xiaojun Wu, Xinjian Xiang, Xianqiang Tang, Yong Zhang

**Affiliations:** 1 School of Automation and Electrical Engineering, Zhejiang University of Science and Technology, Hangzhou, 310023 China; 2 School of Computer Science, Shaanxi Normal University, Xi’an, 710119 China; 3 Zhejiang Kingkind Smart Housing Co., Ltd., Jinhua, 330784 China

**Keywords:** Engineering, Electrical and electronic engineering

## Abstract

To address several common problems of finger vein recognition, a lightweight finger vein recognition algorithm by means of a small sample has been proposed in this study. First of all, a Gabor filter is applied to deal with the images for the purpose of that these processed images can simulate a kind of situation of finger vein at low temperature, such that the generalization ability of the algorithm model can be improved as well. By cutting down the amount of convolutional layers and fully connected layers in VGG-19, a lightweight network can be given. Meanwhile, the activation function of some convolutional layers is replaced to protect the network weight that can be updated successfully. After then, a multi-attention mechanism is introduced to the modified network architecture to result in improving the ability of extracting important features. Finally, a strategy based on transfer learning has been used to reduce the training time in the model training phase. Honestly, it is obvious that the proposed finger vein recognition algorithm has a good performance in recognition accuracy, robustness and speed. The experimental results show that the recognition accuracy can arrive at about 98.45%, which has had better performance in comparison with some existing algorithms.

## Introduction

### Background and motivation

With the development of society, identity recognition technology is increasingly used in our daily life, for example, to perform a payment operation by facial recognition at various shops in China. In contrast to the traditional password mode, biometric recognition technology including both physical and biological characteristics is gradually covering the identity recognition market through its efficient, convenient and secure characteristics. Especially for the physical characteristics, they have been already widely used in everyday life. Facial recognition technology has been widely utilized worldwide at railway stations and airports to make sure that every passenger quickly can pass the gate. Additionally, more and more large banks have begun introducing iris recognition technology to identify customers and help them defend themselves from fake information and unknown risks. In the area of biometric recognition, finger vein recognition has become more and more popular in recent years. It is worth mentioning that finger vein technology has great potential in smart wearable devices and intelligent driving. It is mainly used for identity verification, which can ensure that only authorized personnel can operate the vehicle. On the other hand, finger vein technology can avoid carrying keys, reducing the risk of theft and improving the convenience of trips. There are several reasons why finger vein recognition technology can occupy an important position among different biometric technologies. In comparison with the fingerprints, faces, and other external features of the human body, finger veins cannot be easily faked and misappropriated^1^. The distribution of human veins is different such that everyone is unique^1^. The extraction of the finger vein requires the use of flowing blood, which can ensure that the finger veins can only be collected in vivo^2^. Finger vein collection devices are inexpensive and own powerful potential to be used on a large scale^1,2^.

The principle of image formation for finger veins is to take advantage of the difference in the absorption of light between the deoxyhemoglobin of the vein and other tissues. Usually, a certain wavelength of near-infrared light ( about 700–1000 nm) is used to irradiate the fingers to collect images. The light penetrates the skin and subcutaneous tissue and is scattered therein. Then, the light is absorbed by the deoxyhemoglobin in the venous blood during the scattering process so that the imaging sensor can obtain a dark shadow to present the venous area, and other non-venous areas show high brightness^3^. The collection of finger vein images can be mainly divided into transmission imaging and reflection imaging. The light source and image collector of the former are located at both sides of the finger, and the near-infrared light is used to pass through the finger and irradiate the image collector to obtain the image of the finger vein. The advantage is that the picture obtained in this way is relatively clear. The light source and image collector of the latter are located on the same side of the finger, and near-infrared light is used to penetrate the skin and reflect the image collector to obtain an image of the finger vein. The advantages are that the size and cost of the whole equipment of the collection system can be small and cheap, respectively, but all images can be a little fuzzy^4^.

## Literature review

The main problem of finger vein recognition is that the number and quality of finger vein samples are unsatisfactory. As a kind of new technology, the dataset of finger vein has not been fully developed, hence, the number of finger vein images is extremely limited^5^. Nowadays, finger vein recognition technology can be divided into two types: traditional methods and deep learning methods. The former mainly uses methods including image processing, statistics, and so on, and the latter commonly uses the deep neural network. Bakhtiar et al.^6^ utilized a new texture descriptor called Local Linear Binary Pattern (LLBP) as a finger vein feature extraction technique. The neighborhood shape in LLBP was a straight line, not a square in the Local Binary Pattern (LBP). The LLBP was superior to the LBP and Local Derivative Pattern (LDP). However, the code length of LLBP was much higher than that of LBP, which was not conducive to the model deployment. Lee et al.^7^ used a modified Gaussian high-pass filter to enhance the finger vein and finger shape, which outperformed the traditional Gabor filter. This method still showed a good recognition performance without any finger-aligning algorithm. However, the modified Gaussian high-pass filter could not catch a good performance under low-temperature conditions compared with the Gabor filter. Vlachos et al.^8^ regionally used the Mumford-Shah model to enhance the vein feature of the finger vein image. They used the local entropy thresholding method to detect the depression area to effectively emphasize the section of the finger vein in the image. Ma et al.^9^ used the Pyramid Local Phase Quantization Histogram (PLPQ) to encode the vein image not only in the frequency domain but also between different directions and scales. The experiment proved that the proposed method could effectively improve the ability of finger vein recognition in the system. However, it was still a single-mode recognition method. The performance of the single-mode recognition system was limited, compared with the multi-mode. Shakil et al.^10^ applied the Principal Component Analysis (PCA) to extract finger veins and classify them using the Support Vector Machine (SVM). It combined with the search strategy to match larger patches to avoid searching each pixel. Because it greatly reduced computation time finally. Yang et al.^11^ proposed a finger vein image segmentation based on the combination of the Hessian matrix and the LBF model. This method solved the problem of poor finger vein image segmentation quality caused by uneven light of image, low contrast and edge blur. However, this method still produced some partial discontinuities and isolated points, which affected the final classification effect. To solve the problem of being unable to stably locate the joint, Qiu et al.^12^ proposed a double sliding window model for accurately detecting the location of the joint in the finger vein image. Meanwhile, it reduced the influence of light.

After the year 2015, deep learning has become increasingly popular. Huang et al.^1^ focused on improving the model by using a new attention mechanism, which focused on fine-grained details, and a generalized mean pooling layer, which reduced the dimension of feature maps. Fang et al.^2^ used distal interphalangeal joints to enhance Region of Interest (ROI) positioning and scattering removal to reduce scattering noise in order to focus on image preprocessing, meanwhile, the authors proposed a new multi-scale multiplication rule. In order to cope with the fake finger vein images, Shaheed et al.^13^ proposed a new Deep Separable Convolutional Neural Network (DSCNN) with a Linear Support Vector Machine (LSVM) to automatically detect the true and false finger veins, and computation time and parameters were less than the existing algorithms. To solve the problem of low accuracy of single biometric recognition, Zhou et al.^14^ proposed a dual-modal biometric method based on the feature fusion of finger vein and face, which fused finger vein features and face features into new hybrid features. Then, applying the residual structure to fuse new hybrid features with finger vein features and face features again so as to maximize the retention of biometric information. However, the number of parameters in the MobileNetV2 network, which was a feature extraction module, had not been effectively reduced. Tao et al.^15^ obtained the matching scores for finger vein and finger joint pattern recognition via the Convolutional Neural Network recognition and classification and then obtained the final matching score for the decision via fractional fusion. The recognition rate of this proposed method was higher than the recognition rates of single-finger vein features and single-finger joint pattern features. Yang et al.^16^ proposed a Finger Vein Generative Adversarial Network (FV-GAN) method, which learned from the joint distribution of finger vein images and pattern maps, rather than direct mapping. The FV-GAN adopted a Fully Convolutional Network as the basic architecture, but the fully connected layers were discarded. On the other hand, it relaxed the constraints on the sizes of input images and reduced the computation of feature extraction. Choi et al.^17^ proposed a new method to recover optical fuzzy finger vein images using a modified Conditional Generative Adversarial Network (Conditional GAN) and a Deep Convolutional Neural Network (DCNN) to identify the recovered finger vein images. However, the recovery effects of this method on the darker finger vein area were very poor.

## Method overview and article structure

In this paper, data enhancement methods are used to simulate finger vein images under low temperature, skew and low light conditions, which can enrich sample diversity. The modified VGG-19 model can reduce the number of parameters and increase attention to specific areas. Firstly, the dataset based on the small sample is preprocessed in this experiment. Gaussian filter and guided filter are used to filter the images, which can enhance the quality of the finger vein images. The data of the finger vein images are amplified by the flip, rotation, translation, brightness adjustment and noise addition, hence, the sample size is greatly increased. On the other hand, the Gabor filter is used to simulate the finger vein images under low temperatures such that the dataset can be enriched to enhance the image diversity of the finger vein. Secondly, several convolution layers and fully connected layers are deleted to make the parametric reduction and structure lightweight. After that, in order to reduce the probability of neuron invalidation, the partial activation functions of the original VGG-19 network are replaced by the Leaky ReLU functions. Similarly, to enhance the attention of the model to key regions, the channel attention mechanism and spatial attention mechanism are added to the feature extraction layer of the modified network. Finally, the modified VGG-19 network is used to carry out the feature extraction and feature classification of the finger vein images.

The article structure is shown as follows: In "Literature review" section, the VGG-19 network and cross-entropy loss function are introduced. In "Method overview and article structure" section, the preprocessing method of the finger vein image and the modified VGG-19 network are described in detail. In "Preliminary knowledge" section, the experimental result and analysis are shown in this section. "Method overview and article structure" and "Preliminary knowledge" section are the most significant content of this paper. In the last section, the conclusion and expectation of future work are given.

## Preliminary knowledge

The VGG network was first proposed by the Visual Geometry Group (VGG) of the University of Oxford, which is a kind of deep convolutional network structure. In the year 2014, the VGG network obtained the first and second place in the location and classification competition of the ImageNet Challenge, respectively. Therefore, the network has a strong ability to address some problems of image location and classification^18^. The loss function of the VGG network is the cross-entropy loss. The cross-entropy loss is usually utilized in deep learning because of its simple structure and ease of calculation, which can enhance the stability and speed of the optimization model.

**Figure 1:**
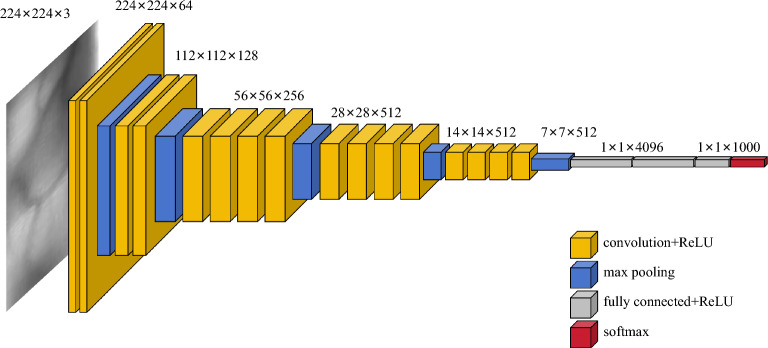
VGG-19 network model.

The VGG network is a kind of deep convolutional network that includes 16 convolution layers, 5 pooling layers, 3 fully connected layers and 1 classification layer. It can provide a good ability for image recognition. The framework of VGG-19 is shown in Fig. 1.

In Fig. 1, each convolutional layer of the VGG-19 network is used to extract features from the finger vein image, because a 3 × 3 convolutional kernel is used with a step size of 1 to fill in the outermost part of the image. Therefore, the size of the image will not be changed after feature extraction. Each convolutional layer is followed by an activation function layer, which uses the ReLU function to nonlinearize the convoluted result and obtain several complicated depth features. The ReLU activation function is shown in Eq. (1):

1 $$f\left( x \right) = \left\{ \begin{gathered} x,x > 0 \hfill \\ 0,x \le 0 \hfill \\ \end{gathered} \right.$$

After the activation function of the 2nd, 4th, 8th, 12th and 16th convolutional layer, each of the above-mentioned layers has a maximum pooling layer, respectively. The size of the filter is 2 × 2 with a step size of 2. The function of this layer is to extract some important features, reduce the image size, and reduce the amount of computation. After the feature map is dealt with by the maximum pooling layer, the length and width have been zoomed out the half of the original image. At the same time, the size of the feature map has been zoomed out the one-quarter of the original image. The classification layer of the VGG-19 network is composed of three fully connected layers. Its input number of the first fully connected layer is dependent on the size and depth of the feature map of the convolutional layer output. Thereby, the input number of the first fully connected layer in the original VGG-19 network is 25,088 (7 × 7 × 512), and the output number is 4096. Both the input number and the output number of the second fully connected layer are 4096. The input number of the third fully connected layer is 4096. Actually, the output number is related to the number of classification categories. If it has 64 categories of image classification problems, the output number of the third fully connected layer should be set to 64.

In comparison with the previous neural networks, the main feature of VGG is that the small convolutional kernel of 3 × 3 has been to replace the large convolution kernels of 5 × 5 and 7 × 7. The advantages of this modification can greatly reduce the number of convolution kernel parameters and increase the network depth. A 1 × 1 image can be obtained by performing two times of the stride = 1, padding = 0 and the convolution kernel being 3 × 3 on a 5 × 5 image. Similarly, a 1 × 1 image can be obtained by performing one time of the stride = 1, padding = 0 and the convolution kernel being 5 × 5 on a 5 × 5 image. The number of convolutional kernels of the former is 18(3 × 3 × 2), and the rear is 25(5 × 5). The number of the former is only 72% of the rear. It is clear that the small convolutional kernel can reduce the number of parameters. A 1 × 1 image can be obtained by performing three times of the stride = 1, padding = 0, and the convolution kernel being 3 × 3 on a 7 × 7 image. Similarly, a 1 × 1 image can be obtained by performing one time of the stride = 1, padding = 0, and the convolution kernel being 7 × 7 on a 7 × 7 image. The number of convolutional kernels of the former is 27(3 × 3 × 3), and the rear is 49(7 × 7). The number of the former is only 55% of the rear. It is obvious that if the old convolutional kernel is bigger, the new small convolutional kernel can reduce both in proportion and number of parameters more.

The 3 × 3 convolution kernel not only increases the depth of the network but also improves the accuracy of the network. Because the 3 × 3 convolution kernel usually needs to be convoluted 2 to 3 times compared with the large convolution kernel that only needs to be convoluted once.

The loss function is applied to calculate the difference between the predicted value *f*(*x*) and the true value Y of the model, which can indicate the direction of model optimization. During the training phase of the model, the loss function calculates the loss value based on the predicted value and true value obtained by forward propagation. After obtaining the loss value, the model updates every parameter through backpropagation to reduce the gap between the real value and the predicted value. Therefore, the model’s predicted value is approaching the direction of the real value to achieve the learning aim. The loss function of the VGG-19 network is the cross-entropy loss function. To target the multi-classification problems, the formula of the cross-entropy loss function is shown in Eq. (2):

2 $$L = \frac{1}{N}\sum\limits_{i} {L_{i} = - } \frac{1}{N}\sum\limits_{i} {\sum\limits_{c = 1}^{M} {y_{ic} } } \log \left( {p_{ic} } \right)$$

where *M* denotes the number of categories; *y*_*ic*_ denotes the symbolic function (0 or 1), the true category of sample *i* is equal to *c* = 1, otherwise, *c* = 0; *P*_*ic*_ denotes the observation sample *i* of the prediction probability *c*.

The cross-entropy loss function has the following characteristics: the value of the cross-entropy loss function is negatively correlated with the prediction result of the model. If the difference between the model prediction result and the true label is small, the value of the loss function is small. However, if the model prediction result is significantly different from the true label, the value of the loss function is large. By minimizing the cross-entropy loss function, the model prediction result can be closer to the true label, and the accuracy and robustness of the model can be enhanced as well.

## Proposed method

The proposed method of this paper is used to improve the image quality and increase the image quantity and diversity, meanwhile, the original VGG-19 model is modified to obtain a smaller, faster and more accurate model. In the preprocessing, a Gaussian filter and a guided filter are used to filter images to achieve the effect of image enhancement. To account for the influence of a small sample, the methods of flip, rotation, translation, brightness adjustment and noise addition are used to perform the data augmentation. In order to reduce the amount of computation, achieve lightweight, avoid neuron invalidation and improve the accuracy of the model, the VGG-19 network is modified as follows: the 12th and 13th convolutional layers and the 1st fully connected layer are deleted totally. The first three activation functions ReLU are replaced by the Leaky ReLU function. Simultaneously, the channel attention mechanism and spatial attention mechanism are added to this modified network.

### ROI extraction

Due to limitations of collection devices and collection environments, many finger vein images are of poor quality, and the existing finger vein database is too small. Both of them may affect the subsequent recognition, therefore, it is necessary to preprocess the finger vein images to enhance the quality and increase the number of finger vein images. The image preprocessing steps mainly include the extraction of ROI, image enhancement and data augmentation.

**Figure 2:**
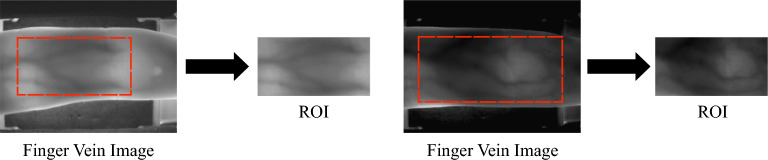
Extraction of ROI.

The extraction of ROI is to retain the feature region and remove the non-feature region, which further improves the recognition ability of the system. After that, it can reduce the image size to facilitate subsequent feature extraction. The extraction of ROI is shown in Fig. 2:

### Image enhancement

Image enhancement reduces image noise and noise interference and strengthens image features, which is conducive to subsequent feature extraction. Image enhancement of this experiment includes a Gaussian filter and a guided filter.

**Figure 3:**
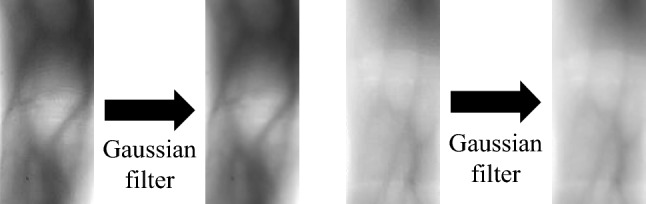
Gaussian filter.

**Figure 4:**
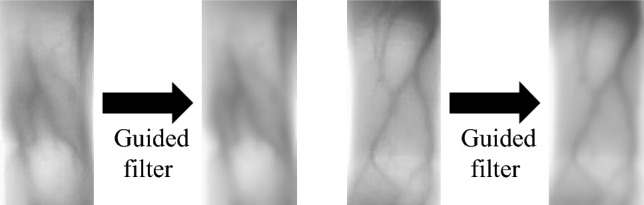
Guided filter.

The Gaussian filter is to filter images with the kernel generated by the two-dimensional Gaussian function. Two parameters need to be set to the standard deviation of the Gaussian kernel and the size of the Gaussian kernel (the size of the kernel is an odd number), respectively. The Gaussian filter can play a role in removing noises and smoothing images, which can effectively enhance the quality of finger vein images. The effect of the Gaussian filter is shown in Fig. 3:

The guided filter is a filtering method that uses the information of the guided image to smooth and enhance the original images^19^. The details of images can be retained extremely when the original images are set as guided images. The guided filter requires filter radius and normalization parameters. Moreover, the guided filter can reduce noise, smooth details and maintain edges, which can effectively enhance the image quality. The effect of the guided filter is shown in Fig. 4:

### Data augmentation

**Figure 5:**
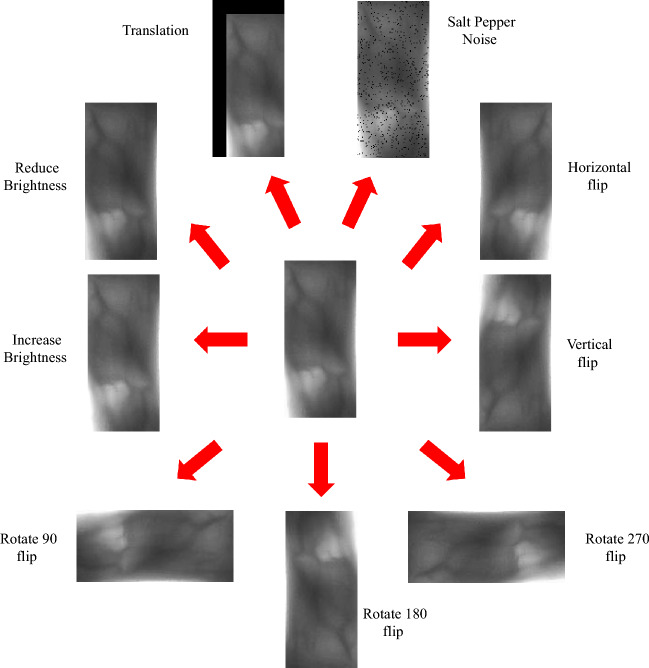
Data augmentation.

Data augmentation is mainly targeted to the lack of data diversity and small samples in finger vein recognition. According to a series of experiments, it is clear that data augmentation for small samples not only can increase the number of samples and increase the diversity of samples but also can enhance the stability and generalization ability, alleviate the problem of overfitting, enhance the robustness and finally enhance the recognition accuracy of the model. Data augmentation in this paper includes the flip, rotation, translation, brightness adjustment and noise addition. It is shown in Fig. 5:

Horizontal flip is the operation of flipping an image along with a vertical central axis. Simply speaking, a horizontal flip can be understood as looking into the mirror. The original image is the real person outside the mirror and the image after the flipping process is the person in the mirror. Hence, a horizontal flip is also known as a mirror flip. Vertical flip is the operation of flipping an image along with a horizontal central axis. Actually, a vertical flip is to transpose the position of the upper half of the image with the bottom half so that the up and down direction of the image can be changed. Consequently, horizontal flip and vertical flip can expand the training dataset and increase the robustness and generalization ability of the model. The central point of an image is the pivot point when it is rotating. In order to increase the training sample and improve the generalization ability of the model, three rotation angles are applied here: 90°, 180° and 270°, respectively. The rotation operation can enrich the training dataset and simulate the deviation of finger placement. Actually, this operation helps enhance the ability to recognize the deviation of finger placement so that it can enhance the model recognition rate. The brightness adjustment operation not only can expand the training dataset but also can simulate the finger vein images under different lighting conditions. The brightness increase can simulate the situation that the background is too bright when the finger vein images are caught, inversely, the brightness reduction can simulate the situation that the near-infrared light is too weak when the finger vein images are caught. These operations can enhance the model generalization ability. Image translation is the operation of moving an image in both horizontal and vertical directions. The translation operation can expand the number of samples, increase the diversity of samples and enhance the robustness and generalization ability of the model. Hence, it can enhance the model's ability to recognize different positions of finger vein images. salt-and-pepper noise refers to the black-and-white noise randomly added to images. Because salt-and-pepper noise, added to images, is beneficial to enhance the robustness and generalization ability of the model.

**Figure 6:**
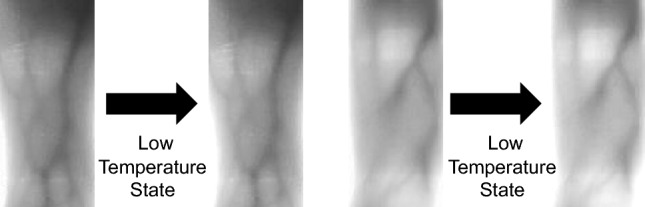
Low temperature.

Low temperature can influence finger vein images, which can increase the brightness of finger vein areas. It can result in a decrease in the difference between the non-finger vein area and the finger vein area, and the difficulty of identification is increased. Therefore, finger vein images under low temperatures are simulated to strengthen the recognition ability. Firstly, it is necessary to set 6 parameters including the filter size, Gaussian kernel standard deviation, angle, wavelength, ellipticity and phase offset. Then, a Gabor filter is created to filter finger vein images to obtain the enhanced images. The enhanced images are converted into grayscale images, after that, the adaptive threshold function is used to binarize the grayscale images into black-and-white images. Meanwhile, the black-and-white images have many independent and small-area noises that must be dealt with by using the opening operation and setting the minimum area threshold to 100. In order to ensure that the main part of the finger vein can be weakened, the black-and-white images are dilated. Finally, finger vein images under the binary mask are extracted and the brightness is improved to achieve the texture-weakening effect. The simulation of low-temperature operation is shown in Fig. 6:

### Feature extraction

**Figure 7:**
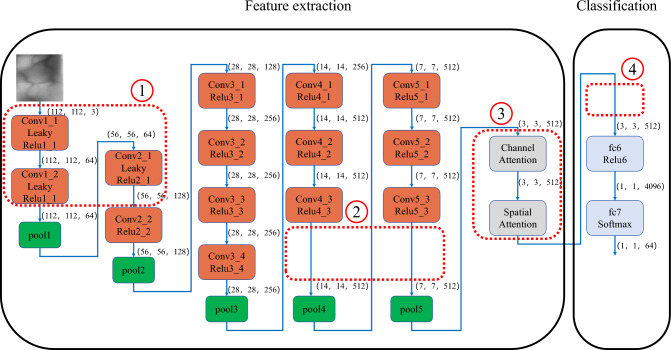
The modified VGG-19 network.

Although the VGG-19 network has the advantages of high accuracy and good recognition ability, its disadvantages are also obvious including the massive computation, neuron invalidation and insufficient attention to certain key regions. These disadvantages affect the final processing speed and accuracy of the model. The modified VGG-19 network is shown in Fig. 7:

In (1) of Fig. 7, three activation functions of the original VGG-19 network are replaced by three Leaky ReLU functions that have a gradient of 0.01 × at *x* < 0. The activation function of the original VGG-19 network is the ReLU function that is equal to itself at *x* > 0 and is equal to 0 at *x* < 0. This situation can cause the negative input to be zeroed in the ReLU, and the neuron cannot be activated by any data such that the ReLU is out of work. Hence, three Leaky ReLU functions are applied in this study, and they are put at the front of the overall network. The Leaky ReLU is shown in Eq. (3):

3 $$f\left( x \right) = \left\{ {\begin{array}{*{20}l} {x,x > 0} \hfill \\ {0.01 \times x,x \le 0} \hfill \\ \end{array} } \right.$$

The VGG-19 network has too many layers, for example, many convolutional layers are used to extract deep information from an image. However, finger vein images are relatively simple, and there is less deep information. Targeting the last 8 convolutional layers with many parameters, two of them can be eliminated through several experiments to achieve the balance of accuracy and computation. Hence, in (2) of Fig. 7, the 12th and 13th convolutional layers are deleted in this study.

**Figure 8:**
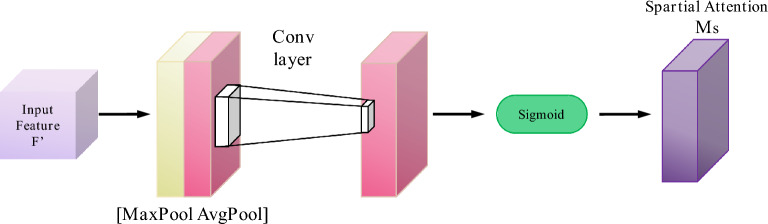
Spatial attention mechanism.

**Figure 9:**
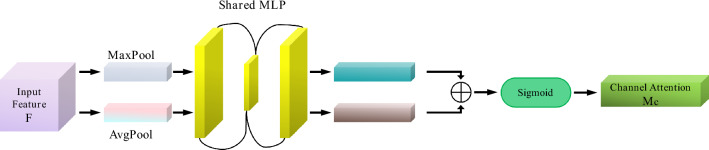
Channel attention mechanism.

In order to improve the model's attention to the important features of finger vein images, the spatial attention mechanism and channel attention mechanism are added to the modified network in (3) of Fig. 7. The attention mechanism can focus on local information to highlight local information. When we look at an image, there is a small part of the content that can attract us certainly, and we focus on this part finally^20,21^. The spatial attention mechanism is to calculate a weight matrix to obtain a weight value that represents the importance of different locations of the input data. This weight matrix can be used as a weighted sum for different locations of the input data. Thereafter, it will allow more attention to the important areas in subsequent processing. The spatial attention mechanism is shown in Fig. 8:

By introducing a spatial attention mechanism, the model can learn the importance of different locations in the data, thereafter, it can process information in a specific area more targeted. This enables the model to better adapt to the difference in the importance of different locations in the finger vein images and enhances the performance and generalization ability of the model. The main function of the channel attention mechanism is to enhance the model's attention to the features among different channels. It can help the model better distinguish and use the information being provided by different channels. The channel attention mechanism is shown in Fig. 9:

By introducing the channel attention mechanism, the model can pay more attention to the important feature channels and enhance the performance of the model.

The three fully connected layers of the VGG-19 network can influence computation greatly. It is well-known that the first fully connected layer contains 4096 nodes, the second one contains 4096 nodes, and the last one contains 1000 nodes. In (4) of Fig. 7, to reduce the computation, the second fully connected layer is deleted here. Because of the good function performance of the cross-entropy loss function, no changes are made to the loss function, and the cross-entropy loss function is still used.

## Experimental results and analysis

### Database

In this study, the finger vein image data is public data which is established by the Tianjin Key Laboratory of Intelligent Signal and Image Processing, referred to as the FV database. The FV database has 64 types of finger samples with 15 finger vein images per finger. Hence, there are only 960 finger vein images in the database. The FV database has been coped with by the ROI extraction to normalize a size of 78 × 170 per image. The sample size is too small, therefore, it has to be amplified in the preprocessing. Finally, a total of 12,480 images of the finger veins are obtained. The training set and test set are divided into 10:3. Thereby, 15 finger vein images are selected from the sample of each type as the test set, and the rest of the finger vein images are selected as the training set. The accuracy of this experiment is expected to exceed that of previous methods.

### Parameter setting

In this paper, the training parameters of ImageNet are used as the pre-training parameters of this modified model by transfer learning training during model training. In this way, the training of the model can be accelerated, and the problem of the small amount of finger vein data can also be appropriately reduced. The input image sizes of the original VGG-19 and ImageNet are 224 × 224, while the finger vein image is only 78 × 170, so the length and width of the input image are set to half of the original. Hence, the size of the image input is set to 112 × 112. The SGD optimizer is used in this experiment. Because the original VGG-19 uses the SGD optimizer, which performed very well without modification. The number of iterations is set to 20 generations. Thanks to the pretraining parameters, the model's accurate value has already reached a high precision after the 20th iteration. The size of the training batch is 64. In fact, the number 64 is a commonly used training batch size, and the size can not only effectively use computing resources, but also maintain stability and efficiency in the model training process. The learning rate is defined as 0.015 in accordance with the parameter adjustment of the experimental process. The Python version is 3.6 (www.python.org/) and the GPU is the NVIDIA GeForce RTX 3060 Laptop GPU.

### Analysis and evaluation

For the classification task in this study, Accuracy is used as the evaluation index of the modified model. The formula for calculating the accuracy rate is shown in Eq. (4):

4 $$Accuracy = \frac{T}{T + F}$$

where *T* is the correct number of recognition and *F* is the number of identification errors.

**Figure 10:**
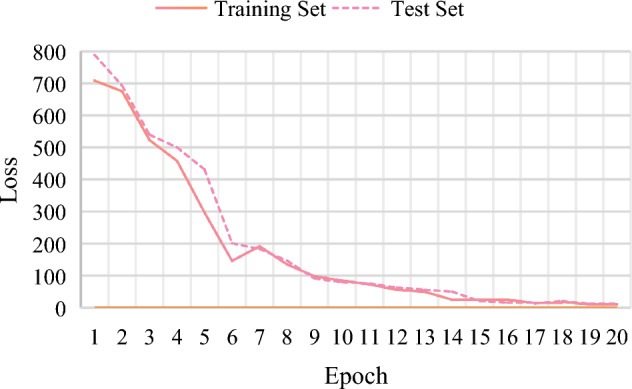
Loss curve.

Figure 10 shows the loss curve of the training process, and the horizontal and vertical coordinates are epoch and loss values, respectively. It is obvious that the curve of the loss function is approaching near zero finally.

**Figure 11:**
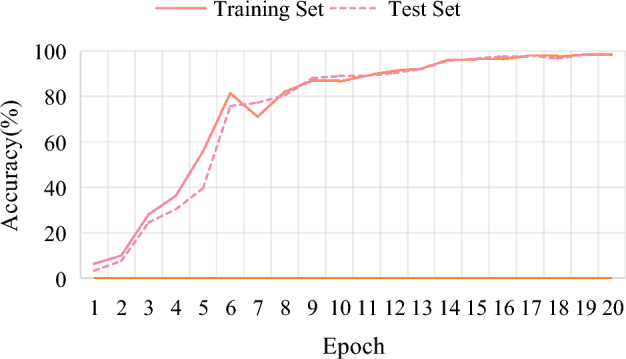
Accuracy curve.

The accuracy curve of the training process is shown in Fig. 11. The values of epoch and accuracy are represented in the horizontal and vertical coordinates, respectively.

The recognition of finger vein images only needs to determine which category the image belongs to, and there is no concept of negative examples. Hence, Recall and mAP evaluation indexes that need to target certain negative examples are not suitable for the recognition of finger vein images.

The modified model is trained for a total of 20 rounds, and the learning rate is 0.015. In 20 rounds of training, the Accuracy value shows an overall upward trend, and the Loss value shows a downward trend. Indeed, it satisfies the designed expectation. The final Accuracy value arrives at about 98.45%. It can greatly indicate that the modified model is suitable for the recognition of finger vein images. However, there is an abnormal situation in the 7th round, because the Accuracy value and Loss value do not follow the general trend. The possible issue is the learning rate setting because a fixed learning rate is applied throughout the whole training process. The fixed learning rate is relatively rigid. It means that the failure to adapt to the update of different parameters can also lead to large fluctuations that affect the final training results.

A series of ablation experiments have been carried out in this study to better understand the influence of each attention mechanism on the detection effect. In the case of the same parameters, a VGG-19 network with only layers’ deletion and activation functions’ substitution is used as the basic comparison network. The results of ablation experiments are shown in Table 1.

**Table 1:** Results of the ablation experiment.

Group	Channel attention	Spatial attention	Accuracy (%)	Loss
A	×	×	96.11	24.85
B	√	×	98.05	13.61
C	×	√	97.47	17.94
D	√	√	98.45	10.47

In Table 1, Group A represents the VGG-19 performing certain operations of the deletion of layers and substitution of activation functions. Group B represents the channel attention mechanism being added to the model based on Group A. Similarly, Group C represents the spatial attention mechanism being added to Group A. Group D represents both the channel attention mechanism and spatial attention mechanism being added to Group A. The ablation experiments show that the recognition accuracy of Group B, Group C, and Group D is higher than that of Group A, which increases by 2.01%, 1.41% and 2.43%, respectively. Consequently, it is obvious that the addition of the channel attention mechanism and spatial attention mechanism can make the network model learn the features from the feature graph more efficiently. The recognition accuracy of Group D is higher than that of Group B and Group C, which proves that the multi-attention mechanism can enhance the recognition accuracy of finger vein images more than the single-attention mechanism.

To further prove the effectiveness and superiority of the modified VGG-19 network, AlexNet, ResNet and GoogLeNet, three classic networks, are chosen to compare with the proposed method under the same experimental environment and model parameters in this paper. The accuracy is used as the evaluation index, and the results are shown in Table 2.

**Table 2:** Results of the comparative experiment.

Group	AlexNet	ResNet	GoogLeNet	VGG-19	Modified VGG-19
Accuracy (%)	74.98	91.08	86.35	95.87	98.45
Loss	182.72	118.01	142.75	54.76	10.47

In Table 2, it is obvious that the loss is negatively correlated with accuracy. The recognition accuracy of the network is higher, and the loss function is smaller. Inversely, the recognition accuracy of the network is lower, and the loss function is larger. The recognition accuracy of AlexNet, ResNet, and GoogLeNet is much lower than the modified VGG-19, hence, the loss function values of the above-mentioned three networks are much higher than our modified network’s value. The recognition accuracy of the modified VGG-19 network is much higher than the other three networks in Table 2, which is improved by 31.3%, 8.09%, 14.01% and 2.69%, respectively. In Table 3, it can be seen that the modified VGG-19 model is used to test the FV-USM and SDUMLA as well, and the accuracy of the test set can reach 96.44% and 96.70%, respectively. Consequently, the modified VGG-19 network is more suitable for finger vein recognition.

**Table 3:** Results of FV-USM, SDUMLA and FV under the modified VGG-19.

Dataset	FV-USM	SDUMLA	FV
	Accuracy (%)	Accuracy (%)	Accuracy (%)
Modified VGG-19	96.44	96.70	98.45

## Conclusion

To address the problem of a small sample in this paper, the amount of finger vein images has been obviously increased by several methods of flipping, rotating, translating, adjusting brightness, adding noise and adding filters. Low temperature can reduce the difference between the finger vein areas and the non-finger vein areas. Hence, we try to simulate a kind of situation of the finger veins at low temperatures for the purpose of looking as a part of the data enhancement. The dataset of finger vein images can support the modified VGG-19 training after data augmentation. In feature extraction and recognition of finger vein images, a modified VGG19 network is proposed in this paper to reduce systemic computation, avoid neuron invalidation, and improve the accuracy of finger vein recognition, respectively. The experimental results show that the recognition accuracy is improved totally in comparison with the original network.

In future work, we will plan to use a Generative Adversarial Network (GAN) for data enhancement of finger vein images. Secondly, we will continue to optimize the network model so as to reduce the internal storage, improve the model operation speed and enhance the model recognition accuracy.

## Data Availability

All original data used during this study can be downloaded on the Internet. The link of the original dataset: https://pan.baidu.com/s/1AleCGmTuaLPBlhmLVXp2FA?pwd=6415. In addition, the available data generated during the current study also can be obtained from the corresponding author on reasonable request.
